# ICAM-1 and ICAM-2 Are Differentially Expressed and Up-Regulated on Inflamed Pulmonary Epithelium, but Neither ICAM-2 nor LFA-1: ICAM-1 Are Required for Neutrophil Migration Into the Airways *In Vivo*

**DOI:** 10.3389/fimmu.2021.691957

**Published:** 2021-08-16

**Authors:** Deborah L. W. Chong, Carine Rebeyrol, Ricardo J. José, Andrew E. Williams, Jeremy S. Brown, Chris J. Scotton, Joanna C. Porter

**Affiliations:** ^1^Centre for Inflammation and Tissue Repair, Division of Medicine, University College London, London, United Kingdom; ^2^Institute of Biomedical and Clinical Sciences, College of Medicine & Health, Exeter, United Kingdom

**Keywords:** epithelium, leukocytes, transmigration, lung, ICAM-2, ICAM-1

## Abstract

Neutrophil migration into the airways is an important process to fight infection and is mediated by cell adhesion molecules. The intercellular adhesion molecules, ICAM-1 (CD54) and ICAM-2 (CD102) are known ligands for the neutrophil integrins, lymphocyte function associated antigen (LFA)-1 (α_L_β_2_; CD11a/CD18), and macrophage-1 antigen (Mac-1;α_M_β_2_;CD11b/CD18) and are implicated in leukocyte migration into the lung. However, it is ill-defined how neutrophils exit the lung and the role for ICAMs in trans-epithelial migration (TEpM) across the bronchial or alveolar epithelium. We found that human and murine alveolar epithelium expressed ICAM-1, whilst the bronchial epithelium expressed ICAM-2, and both were up-regulated during inflammatory stimulation *in vitro* and in inflammatory lung diseases such as cystic fibrosis. Although β_2_ integrins interacting with ICAM-1 and -2 mediated neutrophil migration across human bronchial epithelium *in vitro*, neither ICAM-2 nor LFA-1 binding of ICAM-1 mediated murine neutrophil migration into the lung or broncho-alveolar space during LPS-induced inflammation *in vivo. *Furthermore, TEpM of neutrophils themselves resulted in increased epithelial junctional permeability and reduced barrier function *in vitro*. This suggests that although β_2_ integrins interacting with ICAMs may regulate low levels of neutrophil traffic in healthy lung or early in inflammation when the epithelial barrier is intact; these interactions may be redundant later in inflammation when epithelial junctions are disrupted and no longer limit TEpM.

## Introduction

Neutrophilic inflammation underlies many chronic lung diseases including cystic fibrosis (CF), bronchiectasis, and chronic obstructive pulmonary disease (COPD) (1, 2). During inflammation or infection, the movement of leukocytes such as neutrophils from the blood across the endothelium is a highly-organized process, consisting of well characterized steps of capture, rolling, arrest, adhesion, crawling and finally transmigration of leukocytes across the blood vessel wall (3). Each of these steps is mediated *via* interactions between cell adhesion molecules expressed by leukocytes and endothelial cells, such as integrins, selectins, intercellular adhesion molecules (ICAMs) and junctional adhesion molecules (JAMs) (4).

The role of ICAM-1 (CD54) and ICAM-2 (CD102) in mediating leukocyte trans-endothelial migration has been studied. ICAM-1 is highly expressed on endothelial cells (5) and is required for neutrophil adhesion and trans-endothelial migration (6). ICAM-2 is also expressed on endothelial cells (7) and mediates neutrophil crawling and subsequent extravasation through the endothelium *in vivo* (8). Although we understand the mechanisms of leukocyte trans-endothelial migration in many organs, it is becoming clearer that leukocytes enter the lung interstitium *via* different mechanisms (9, 10). Due to low blood flow velocity and the small size of pulmonary capillaries, neutrophils do not undergo the conventional rolling steps of trans-endothelial migration (11). Therefore, different mechanisms may occur to mediate leukocyte migration into the lung in response to inflammation or infection.

Furthermore, the mechanisms by which leukocytes leave the lung interstitium are unclear. In particular, the mechanisms of trans-epithelial migration (TEpM) at the level of airways and/or alveolar epithelia are ill-defined. In contrast to trans-endothelial migration, leukocytes must cross the epithelial cell barrier in a basolateral-to-luminal direction. Therefore, different cell adhesion molecules may be required compared to trans-endothelial migration. In addition, expression levels of cell adhesion molecules on neutrophils are modulated as these cells move out of the blood, through the interstitium and across the lung epithelium (12). For example, the expression of lymphocyte function-associated antigen (LFA)-1 (α_L_β_2_; CD11a/CD18) on neutrophils decreases as they exit the blood into the airways, whilst macrophage-1 antigen (Mac-1, α_M_β_2_; CD11b/CD18) expression increased upon entering the lung interstitium and decreased after crossing the bronchial epithelium (12). Therefore, neutrophils arriving at the basolateral surface of the epithelium may have altered activation status and different cell adhesion ligands on epithelial cells may be required to mediate TEpM.

Neutrophil TEpM into the bronchial airways and alveolar airspaces is important to clear pathogens but may cause collateral tissue damage (13). In a murine model of *Pseudomonas aeruginosa*-induced pneumonia, neutropenic mice exhibited increased mortality, emphasising the importance of neutrophil recruitment into the lung for bacterial clearance (14). Migration across the bronchial epithelium has also been proposed as a mechanism by which neutrophils and other inflammatory cells are cleared from the interstitium. Eosinophils, unable to migrate across the bronchial epithelium accumulate in the interstitium of ICAM-2 deficient mice during allergic airway inflammation to worsen airway inflammation (15). In contrast, Li et al. showed that preventing neutrophils moving across the alveolar epithelium, by reducing a chemokine gradient, reduced mortality from pulmonary inflammation (16). This suggests that the consequences of leukocyte migration across the bronchial or the alveolar epithelium may vary and be differentially regulated during inflammation.

Despite the exact mechanisms remaining unknown, there is evidence to suggest a role for β2 integrins, such as LFA-1 and Mac-1, interacting with epithelial ligands in mediating neutrophil TEpM *in vitro*. Although there are discrepancies in the literature. Three studies have shown that migration of human neutrophils through monolayers of human airway epithelial cell lines is reduced by pre-treatment with blocking antibodies against ICAM-1 (17–19), which is contrasted by one study showing no effect (20). In addition, we have previously shown that adhesion of the β2 integrin, LFA-1, to ICAM-1 and ICAM-2 on the basolateral surface of the epithelium plays a role in T lymphocyte migration (basal-to-apical) across a tight barrier of bronchial epithelial cells (21). However, it is unclear if these *in vitro* models accurately reflect the animal models of lung inflammation. Therefore, we sought to investigate the expression of ICAM-1 and ICAM-2 on bronchial and alveolar epithelia on normal and inflamed lung and the role of these molecules in mediating neutrophil TEpM *in vitro* and *in vivo*.

## Material and Methods

### Antibodies

Bronchial epithelial cell cultures were incubated with 10 μg/ml of the following functional blocking antibodies: ICAM-1 (clone 15.2, a domain (D)-1 blocking antibody that inhibits LFA-1: ICAM-1 binding, GenTex, USA), ICAM-2 (clone CBR-IC2/2, GenTex), VCAM-1 (clone BBIG-V1, R&D Systems, UK), CD29 (clone P5D2, Santa Cruz biotechnology, USA), CD18 (clone TS1/18, Biolegend, USA), CD11b (clone 2LP19c, Santa Cruz biotechnology), CD49d/VLA-4 (clone HP2/1, GenTex) and CD11a (clone 38, GenTex). Normal mouse IgG1 (Santa Cruz biotechnology), and IgG2 (GenTex) were used as isotype controls.

### Patient Samples, Cells, and Mice

Human neutrophils or lung tissue biopsies from healthy controls, and CF or COPD patients were collected with local written consent and ethical approval from the UK National Research Ethics Committee (13/LO/0900). Human bronchial epithelial cells (HBECs) were cultured from explants and passaged as previously described (22). Human umbilical vein endothelial cells (HUVECs) were purchased from a commercial source (Lonza, UK) and passaged according to supplier’s guidance. 16HBE14o- cells (a human bronchial epithelial cell line) were cultured in αMEM supplemented with 10% FBS, 2 mM L-glutamine, and 1% penicillin/streptomycin (ThermoFisher Scientific, UK). All epithelial cells were routinely screen for mycoplasma contamination by PCR. All animal studies were ethically reviewed and conducted in line with UK Home Office Regulations. C57BL/6 ICAM-2^+/+^ (WT) and ICAM-2^-/-^ (KO) mice (15) were housed and maintained at University College London, with accordance to UK Home Office Regulations.

### Immunohistochemistry and Immunofluorescence Staining

Paraformaldehyde (PFA) fixed human or murine lung biopsies were embedded in paraffin wax and 5 μm sections cut for IHC or IF staining. Heat-induced epitope retrieval with citrate buffer, pH 6.0 or Tris-EDTA buffer, pH 9.0 was performed with murine lung sections prior to blocking with 4% goat serum/1% BSA/PBS and staining with ICAM-1 (clone YN1/1.7.4, ThermoFisher Scientific), ICAM-2 (clone EPR18231-183, Abcam, UK) or rat or rabbit IgG isotype control (Abcam) antibodies. Human lung sections were stained with ICAM-1 (clone 15.2, ThermoFisher Scientific), ICAM-2 (clone CBR-IC2/2, ThermoFisher Scientific) or CK5 (clone 34bE12, Abcam) antibodies. For IHC studies, sections were developed using HRP-conjugated secondary antibodies, NovaRED Peroxide (HRP) substrate kit (Vector Laboratories, UK) and counter-stained with haematoxylin using an automated slide stainer (Sakura Tissue-Tek DRS 2000). For IF studies, fluorescently-conjugated secondary antibodies were used, followed by nuclear DAPI staining. IHC sections were scanned on a Nanozoomer Digital Slide Scanner and images analysed using NDP.view software (Hamamatsu Corporation, Japan). IF sections were analysed using a AxioScan Z1 microscope and Zen-Lite software (Zeiss, Germany).

### Bronchial Epithelial Air-Liquid Interface Model

Trans-well permeable inserts with 5 μm pores (Millipore, UK) were pre-coated overnight with 250 μg/ml type IV collagen (Sigma-Aldrich, UK). The following day, trypsinized 16HBE14o- cells were diluted to a concentration of 1x10^6^ cells/ml and stained with Vybrant DiD fluorescent cell labelling dye (ThermoFisher Scientific) for 20 min at 37°C. Cells were washed three times with αMEM and resuspended at 1.5x10^6^ cells/ml. 75 μl of cell suspension was seeded on to inverted trans-wells and allowed to adhere for 4 h at 37°C, 5% CO_2_. Trans-wells were loaded into 24-well receiver plates with 900 µl of growth medium in the outer well (apical side of epithelium) and 200 µl in the insert of the trans-well (basal side of the epithelium). Medium was replaced every 2 days. After 6 days expansion, cells were transferred to ALI culture, by rinsing trans-wells with PBS and adding 300 µL αMEM to the apical side. 16HBE cells were cultured in ALI for 7 days. Unstained HBECs were also seeded on to trans-well as described above and were cultured at ALI for 21 days. In later experiments, epithelial cells were pre-treated with 50 ng/ml TNF-α (R&D systems) for 24 h.

### Quantitative PCR

Total RNA was extracted from epithelial cells using acidic phenol method with chloroform and TRIzol (Invitrogen, UK) prior to conversion into cDNA with qScript Reverse Transcriptase (Quantabio, USA). qPCR was performed with Taqman pre-designed ICAM-1 and ICAM-2 probes and Sybr-green (Applied Biosystems, UK) using an Eppendorf real-plex Mastercycler PCR machine (Eppendorf, USA). All samples were run in duplicate and fold change in gene expression was determined based on ΔCt calculations compared against β2-microglobulin and GAPDH reference genes as controls.

### Neutrophil Isolation and TEpM Assay

Human neutrophils from peripheral blood from healthy controls were isolated by dextran/saline red blood cell sedimentation, followed by gradient centrifugation using Percoll (Sigma-Aldrich) and erythrocyte hypotonic lysis. Neutrophils were labelled with 1 μM CMFDA fluorescent dye (ThermoFisher Scientific) in HBSS without Ca^2+^ and Mg^2+^ (HBSS-) for 30 min at 37°C. Cells were washed in HBSS- and resuspended at 5x10^6^ cells/ml in HBSS with Ca^2+^ and Mg^2+^ (HBSS+). Bronchial epithelial ALI cultures were washed in HBSS+ prior to use. To assess neutrophil TEpM, 100 ng formylmethionyl-leucyl-phenylalanine (fMLP; Sigma-Aldrich) in HBSS+ was added to the outer well and 5x10^5^ labelled neutrophils added to the trans-well. Neutrophils were left to migrate for 3 h at 37°C, 5% CO_2_. In inhibition studies, neutrophils were pre-treated at 4°C with 10 μg/ml of blocking antibodies, isotype control antibodies or HBSS- for 30 min. Blocking antibodies against cell adhesion molecules expressed on bronchial epithelial cells were add to the top of the trans-well (basolateral side) for 30 min before washing with HBSS+ and neutrophils added to the trans-well for TEpM. After neutrophil migration, to detach transmigrated neutrophils adherent to the epithelial layer, 30 μl 0.5 M EDTA was added to each well and incubated for 10 min at 4°C. Detached cells in the outer wells were removed and rinsed with HBSS+ prior to fixation with 2% PFA and CMFDA^+^ Vybrant DiD^-^ neutrophils were counted using a BD Bioscience FACSVerse flow cytometer (BD Biosciences, UK) and analysed with FlowJo Version 10 software (FlowJo, USA) (**Supplemental Figure 1** for gating strategy). The percentage of transmigrated neutrophils was calculated relative to the number of unstimulated neutrophils that had migrated through untreated monolayers towards fMLP (control group).

### Mouse Models of LPS-Induced Sterile Inflammation or Pneumococcal Infection

8-14 week old male and female C57BL/6 ICAM-2^+/+^ (WT) or ICAM-2^-/-^ (KO) mice were used for *in vivo* studies. To induce sterile inflammation in the lung, mice were randomised into 2 treatment groups and given 3.3 μg/mouse LPS (Sigma-Aldrich) in a volume of 50 μl PBS or PBS only *via* an intra-nasal route. Mice were monitored for disease severity and culled at an established humane endpoint. Broncho-alveolar lavage fluid (BALF) was obtained from 3 washes of 1 ml PBS and centrifuged for 5 min at 300 *g*. BALF cells were counted by cyto-centrifugation. Lungs were harvested and homogenized in PBS to obtain a single cell suspension before erythrocyte hypotonic lysis and staining with Ly6G-APC-Cy7 (clone 1A8), Ly6C-V450 (clone AL-21), CD11b-PerCP-5.5 (clone M1/70), CD11c-PE-Cy7 (clone HL3), MHCII-PE (clone M5/114.15.2) (BD Biosciences) or F4/80-APC (clone C1:A3-1; BioRad, UK) antibodies. Cells were fixed in 2% PFA and read on a flow cytometer and analysed using FlowJo software (**Supplemental Figure 2** for gating strategy). For pneumococcal infection experiments, mice were given 3-5x10^6^ CFU *Streptococcus pneumoniae* 19F isolate *via* an intranasal route. Cellular recruitment into the BALF or lung was quantified as described above (**Supplemental Figure 3**). For *in vivo* ICAM-1 blocking studies, mice were given 2 doses of 3 mg/kg ICAM-1 blocking (clone KAT-1, a D-1 blocking antibody) or IgG2a isotype control antibody (Biolegend) (23) *via* intra-peritoneal route 1 day prior and on the day of LPS or pneumococcal challenge.

### Trans-Epithelial Electrical Resistance and Cell Permeability

TEER and cell permeability to sodium or dextran conjugated with fluorescein was assessed in bronchial cell monolayers. TEER measurements were conducted using an STX2 electrode and EVOM2 meter (World Precision Instruments, USA). The STX2 electrode was equilibrated in HBSS- prior to TEER values taken by a 4-point measurement system on trans-well inserts. Type IV collagen coated inserts without cells were used as background controls. Permeability to 100 μg/ml of sodium-fluorescein (376.27 Da) or 250 µg/ml of fluorescein-dextran (3 KDa) solutions (both from Sigma-Aldrich) was assessed by loading 200 μl fluorescein conjugated molecules into the apical side of the insert and allowing to permeate to the basolateral side from 30 min to 3 h at 37°C, 5% CO_2_. 100 μl samples were collected from the basolateral side for fluorometric analysis using 485nm excitation and 590nm emission filters. Concentrations were obtained from a standard curve with diluted fluorescein conjugated solution. Permeability was determined using the equation: (dQ/dt)/C0*A), where dQ/dt is the rate of permeation (µg/sec), C0 is the initial concentration (µg/ml) and A is surface area of the monolayer (cm^2^).

### Statistics

Data analysis was performed using GraphPad Prism V9 (GraphPad software Inc, USA). Data are presented as mean ± standard error of mean (SEM) of 3 independent experiments, performed in triplicate unless otherwise stated. Statistical analysis was performed as stated in figure legends. Differences between variables were considered statistically significant for *p*-values < 0.05 and are represented as follows: n/s = not significant, *p < 0.05, **p < 0.01 and ***p < 0.001.

## Results

### ICAM-1 and ICAM-2 Are Differentially Expressed on Healthy Lung Epithelium

**Figure 1:**
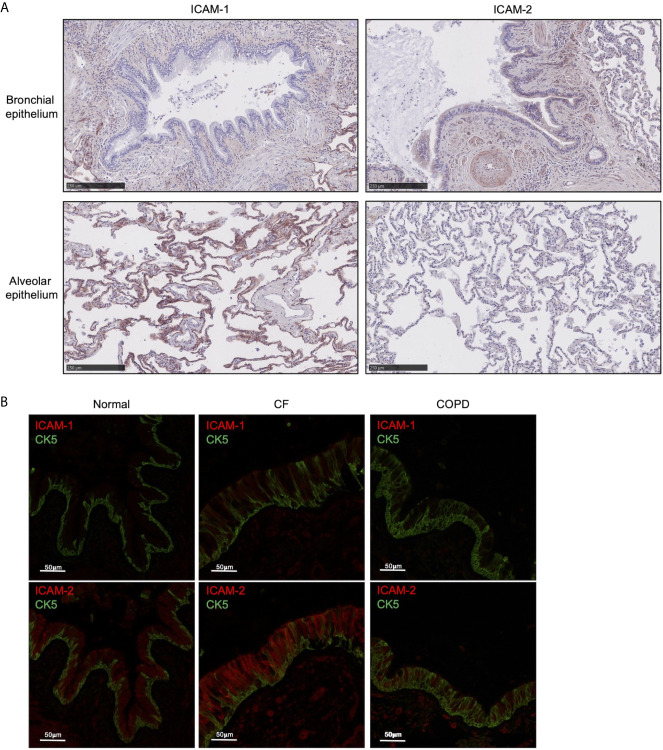
ICAM-2 is expressed on healthy human bronchial epithelium and is up-regulated in inflamed diseased airways. **(A)** ICAM-1 or ICAM-2 expression (red) was visualized on bronchial (top row) or alveolar (bottom row) epithelium in human normal lung tissue by immunohistochemistry. **(B)** ICAM-1 (red, top row) or ICAM-2 expression (red, bottom row) was visualized on CK5^+^ epithelium (green) by immunofluorescent staining in human normal lung or diseased CF or COPD lung tissue. Representative images from 3 normal, 3 CF and 3 COPD lung donors are shown and size is denoted by scale bar.

The mechanisms by which neutrophils transmigrate across inflamed airway epithelium are ill-defined and the role of ICAM-1 and ICAM-2 in mediating neutrophil TEpM is unknown. We initially examined human lungs for ICAM-1 and ICAM-2 expression on the epithelium. Immunohistochemical analysis of healthy adult human lungs revealed that ICAM-1 was primarily expressed on the alveolar epithelium and only mildly on the bronchial epithelium (**Figure 1A**). ICAM-2 was strongly expressed on the bronchial epithelium and weakly on the alveolar epithelium (**Figure 1A**), indicating that ICAM-1 and ICAM-2 are differentially expressed on healthy adult human lung epithelium.

### ICAM-2 Is Up-Regulated on the Bronchial Epithelium During Neutrophilic Inflammatory Lung Diseases

We next examined whether inflammatory conditions could affect ICAM-1 or ICAM-2 expression on the bronchial epithelium. Normal human adult lungs expressed much lower levels of ICAM-1 than ICAM-2 on the bronchial epithelium as marked by CK5 positive staining (**Figure 1B**). Interestingly, there was striking up-regulation of ICAM-2 expression in the airways from patients with CF (a genetic disease characterized by neutrophilic-driven lung inflammation) compared to normal lung (**Figure 1B**). ICAM-1 expression was moderately increased on CF bronchial epithelium compared to normal lung (**Figure 1B**). However, only modestly increased levels of ICAM-1 and ICAM-2 expression were found on inflamed bronchial epithelium from patients with COPD (another lung disease characterized with neutrophilic-driven inflammation) compared to normal lung (**Figure 1B**). These observations indicated that ICAM-1 and ICAM-2 expression may be up-regulated during certain inflammatory lung diseases that are associated with airway neutrophilia.

### Bronchial Epithelial Cells Grown at ALI Showed Up-Regulation of ICAM-2 Expression Upon Stimulation With TNF-α

**Figure 2:**
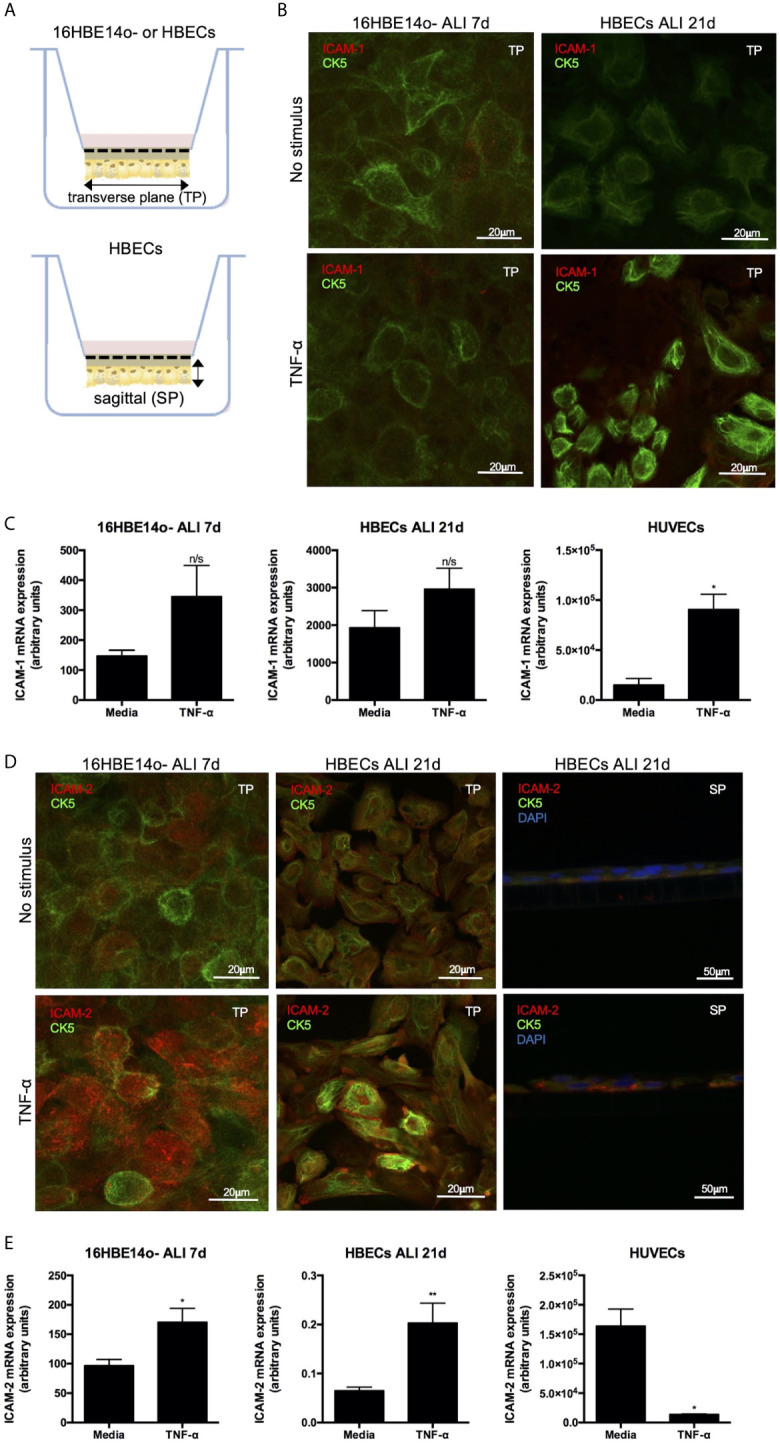
ICAM-2 expression is up-regulated on bronchial epithelial cells following TNF-α stimulation *in vitro*. **(A)** Schematic of 16HBE14o- or HBECs monolayers grown at ALI using a trans-well *in vitro* system. Solid arrows denote transverse (TP) or sagittal planes (SP) used for sectioning for later immunofluorescence studies. **(B)** Immunofluorescent staining of ICAM-1 expression (red) on unstimulated or TNF-α stimulated CK5^+^ (green) 16HBE14o- or HBECs grown at ALI. Representative images are shown on the TP and image size is denoted by the scale bar. **(C)** ICAM-1 mRNA expression was determined in media (*n*=5 different passage) or TNF-α stimulated (*n*=5-6 different passages) 16HBE14o- or HBECs grown at ALI or HUVECs by qPCR. Fold change in ICAM-1 gene expression was normalised to 2 reference genes. **(D)** Immunofluorescent staining of ICAM-2 expression (red) was determined on unstimulated or TNF-α stimulated CK5^+^ (green) 16HBE14o- or HBECs grown at ALI. Representative images are shown on the TP or SP. Additional DAPI nuclear staining (blue) was performed on sections on the SP. Image size is denoted by the scale bar. **(E)** ICAM-2 mRNA expression was determined in media (*n*=5-7 different passages) or TNF-α stimulated (*n*=4-6 different passages) 16HBE14o- or HBECs grown at ALI or HUVECs by qPCR. Fold change in ICAM-2 gene expression was normalised to 2 reference genes. All statistical testing was performed using paired t-test and any differences are indicated (n/s, not significant, *p < 0.05, **p < 0.01).

As ICAM-2 and ICAM-1 expression were increased on lung epithelium from patients with an inflammatory lung disease associated with airway neutrophilia, we next examined whether these cell adhesion ligands mediate neutrophil TEpM. To model neutrophil TEpM in a physiological basal-apical direction, an *in vitro* model system in which epithelial cells were grown on the undersurface of a trans-well insert was used (**Figure 2A**). The distribution of ICAMs in monolayers of a normal bronchial epithelial cell line, 16HBE14o- (16HBE) or primary human lung bronchial epithelial cells (HBECs) was assessed. Similar to our previous observations, unstimulated 16HBE or HBECs had low ICAM-1 expression (**Figure 2B**), which was modestly increased after TNF-α stimulation of HBECs (**Figure 2B**). ICAM-1 mRNA was also detected in unstimulated 16HBE or HBECs by qPCR analysis, however these levels were ~7-30 fold lower than in unstimulated human umbilical vein endothelial cells (HUVECs) (**Figure 2C**). HUVECs treated with TNF-α lead to a significant increase in ICAM-1 mRNA expression (**Figure 2C**) consistent with previous data (24). In contrast, ICAM-1 mRNA expression in TNF-α stimulated 16HBE or HBECs did not significantly differ compared to media treated controls (**Figure 2C**).

Unstimulated 16HBE or HBECs expressed basal levels of ICAM-2 protein, which was dramatically increased upon TNF-α stimulation (**Figure 2D**). In agreement with the previous data (25), TNF-α stimulated HUVECs exhibited significantly decreased expression of ICAM-2 mRNA compared to unstimulated cells (**Figure 2E**). TNF-α stimulation of 16HBE or HBECs lead to a significant increase in ICAM-2 mRNA levels compared to unstimulated controls (**Figure 2E**), which supports our immunofluorescence studies showing increased ICAM-2 protein under these conditions. This demonstrated that whilst TNF-α treatment only had mild effects on ICAM-1 mRNA or protein expression in bronchial epithelial cells, this pro-inflammatory mediator had differential effects on ICAM-2 expression, leading to significant up-regulation of protein and mRNA expression on bronchial epithelial cells.

### Neutrophil Migration Across Bronchial Epithelial Cells Required β2 Integrins and ICAM-1, but Not ICAM-2

**Figure 3:**
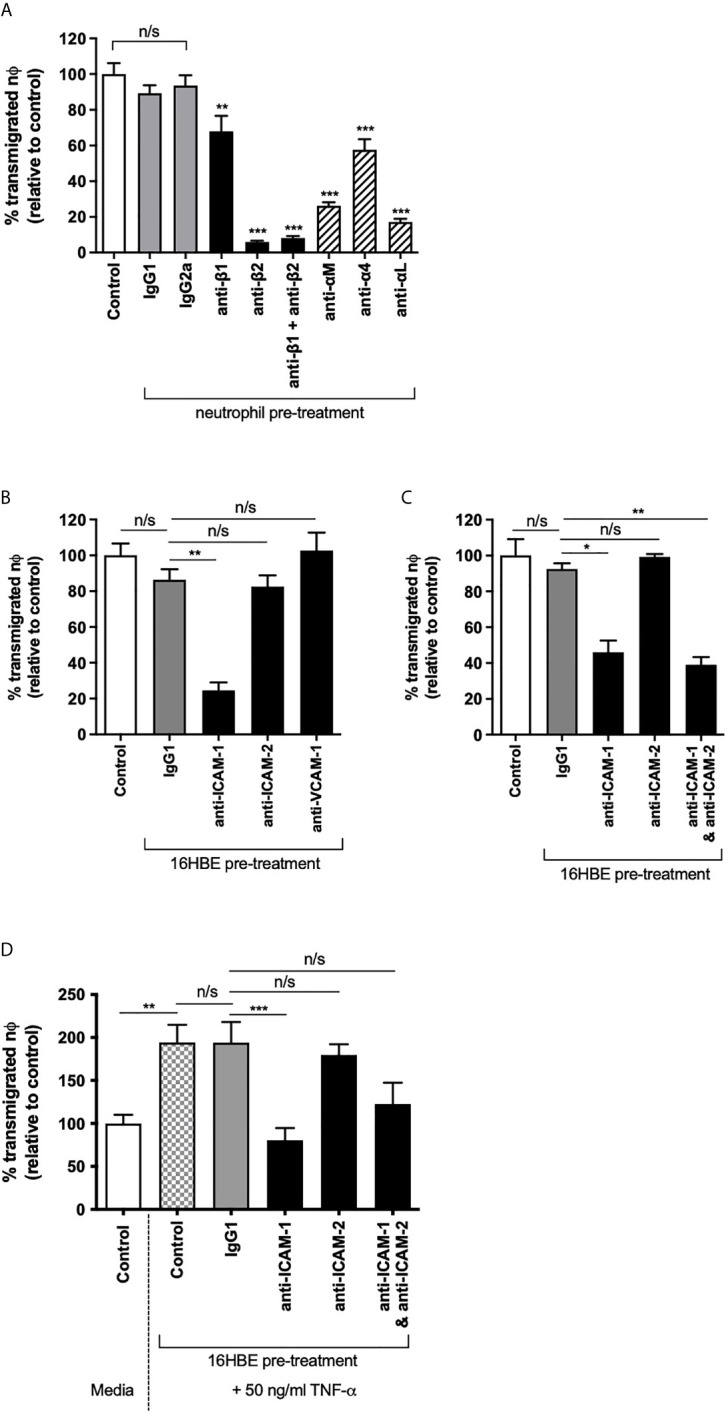
Neutrophil trans-epithelial migration towards fMLP required β2 integrins and ICAM-1 but not ICAM-2. **(A)** Neutrophil TEpM through 16HBE14o- ALI monolayers were assessed using media, blocking antibodies (anti-β1, β2, αM, α4 or αL) or matched IgG1 or IgG2a isotype controls pre-treated neutrophils (*n*=3 donors). Percentage of transmigrated neutrophils was calculated relative to transmigrated unstimulated neutrophils (control). **(B)** Neutrophil TEpM (*n*=3 donors) was assessed through 16HBE14o- ALI monolayers pre-treated on the basolateral side with IgG1 isotype control or blocking antibodies against ICAM-1, ICAM-2 or VCAM-1. Percentage of transmigrated neutrophils was calculated relative to migrated neutrophils through unstimulated monolayers (control). **(C)** Neutrophil TEpM (*n*=3 donors) was assessed through 16HBE14o- ALI monolayers pre-treated on the basolateral side with media, IgG1 isotype control or blocking antibodies against ICAM-1, ICAM-2 or both together. Percentage of transmigrated neutrophils was calculated relative to migrated neutrophils through unstimulated monolayers (control). **(D)** 16HBE14o- ALI monolayers were pre-treated with media or 50 ng/ml TNF-α for 24 h. The basolateral side of the monolayers were then either treated with media, IgG1 isotype control or blocking antibodies against ICAM-1, ICAM-2 or both. Neutrophil TEpM (*n*=3 donors) was assessed relative to migrated neutrophils through unstimulated monolayers (control). Any statistical differences were determined using 1-way ANOVA with Holm-Sidak post-hoc correction (n/s, not significant, *p < 0.05, **p < 0.01, ***p < 0.001). Asterisks above bars show comparisons to matched IgG controls, unless stated otherwise.

The role of ICAM-2 in human neutrophil TEpM was next assessed in response to the recognized neutrophil chemoattractant, fMLP (26). Pre-treating human neutrophils with blocking antibodies against β1 or β2 integrins showed that TEpM through a tight monolayer of 16HBE was mediated primarily through β2 integrins, as β2 integrin blockade significantly reduced neutrophil TEpM compared to the matched isotype control treated cells (**Figure 3A**). Combined β1 and β2 integrin blockade on neutrophils did not further reduce neutrophil TEpM compared to β2 integrin only inhibition, strongly supporting the role of β2 integrins in mediating neutrophil transmigration (**Figure 3A**). Specifically, TEpM was dependent on αLβ2 (LFA-1) and αMβ2 (Mac-1) expressed on neutrophils, both integrins that can bind ICAM-1 and ICAM-2 (**Figure 3A**). To identify the basolateral receptor for these integrins, bronchial epithelial cell monolayers were pre-treated on the basal side with blocking antibodies against cell adhesion ligands. ICAM-1 blockade, with a D-1 antibody that specifically inhibits LFA-1: ICAM-1 binding, significantly decreased neutrophil TEpM, whereas ICAM-2 or VCAM-1 (CD106; another cell adhesion molecule expressed on epithelial cells) blockade had no effect on neutrophil TEpM compared to isotype control treated cells (**Figure 3B**). This was surprising as unstimulated bronchial epithelial cells exhibited low ICAM-1 expression compared to much higher levels of ICAM-2. ICAM-1 and ICAM-2 combined blockade did not further reduce neutrophil TEpM compared to ICAM-1 only inhibition suggesting that ICAM-2 was redundant in mediating neutrophil migration *in vitro* (**Figure 3C**). To ensure ICAM-2 expression was maximized on bronchial epithelial cells, monolayers were pre-stimulated with TNF-α, and under these conditions neutrophil TEpM was still dependent on ICAM-1 with no role for ICAM-2 in this neutrophil TEpM *in vitro* model (**Figure 3D**).

### Neutrophil TEpM Affects Bronchial Epithelial Integrity

**Figure 4:**
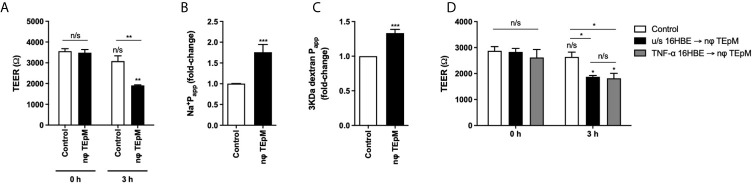
Neutrophil transmigration affects bronchial epithelial cell integrity. **(A)** TEER or permeability to **(B)** sodium-fluorescein or **(C)** 3 KDa Dextran-FITC was measured before (0 h) and after 3 h of neutrophil TEpM (nφ TEpM) through 16HBE14o- ALI cultures. Wells with fMLP and without neutrophils were used as a control group. **(D)** TEER was measured before (0 h) and after 3 h neutrophil TEpM through either unstimulated 16HBE14o- ALI cultures (u/s 16HBE → nφ TEpM) or TNF-α stimulated 16HBE14o- ALI cultures (TNF-α 16HBE → nφ TEpM). Statistical testing was performed using 2-way ANOVA with Holm-Sidak post-hoc correction or unpaired student t-test (n/s, not significant, *p < 0.05, **p < 0.01, ***p < 0.001). Asterisks above bars show comparisons to 0 h controls, unless stated otherwise.

As ICAM-1 significantly contributed to neutrophil TEpM *in vitro*, we next investigated the integrity of the bronchial epithelial cell monolayers by measuring epithelial permeability before and after neutrophil TEpM. Untreated bronchial epithelial cell monolayers had a high trans-epithelial resistance (TEER) of 3487 Ω ± 152.9 Ω, however after 3 h of neutrophil TEpM, the TEER fell significantly to 1912.3 Ω ± 27.9 Ω compared to untreated monolayers (**Figure 4A**). The permeability to Na^+^ ions and 3 KDa dextran also increased significantly by 75% and 33% respectively compared to untreated monolayers (**Figures 4B, C**). Furthermore, when bronchial epithelial cell monolayers were pre-treated with 50 ng/ml TNF-α before neutrophil TEpM, the TEER was not significantly different compared to unstimulated bronchial epithelial cell monolayers following neutrophil TEpM (**Figure 4D**). This demonstrated that neutrophils themselves significantly increase the permeability of either unstimulated or TNF-α treated epithelial monolayers as they move across but the tight junctions in these fully differentiated monolayer of cells are still maintained as the TEER was approximately 2000 Ω.

### ICAM-1 and ICAM-2 Are Differentially Expressed on Naïve Wild-Type Murine Lung Epithelium

**Figure 5:**
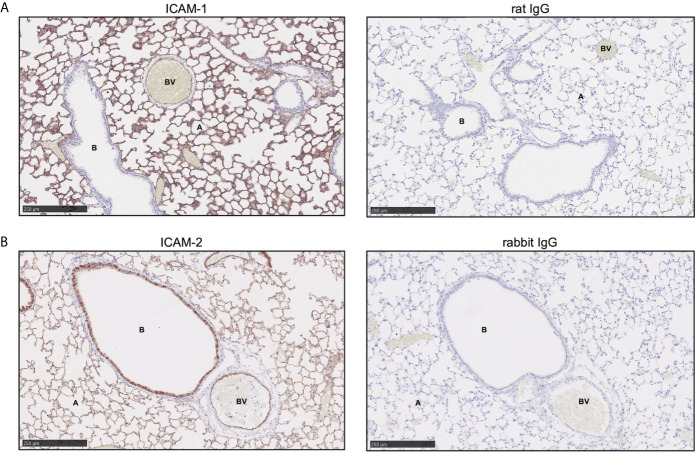
ICAM-1 and ICAM-2 are differentially expressed on wild-type murine lung epithelium. **(A)** ICAM-1 expression (red) was visualized in wild-type murine normal lung tissue by immunohistochemistry. **(B)** ICAM-2 expression (red) was visualized in wild-type murine normal lung tissue by immunohistochemistry. Minimal non-specific primary antibody binding was detected by staining with matched IgG isotype control antibody. Representative images are shown and size is denoted by the scale bar (A, alveoli; B, bronchiole; BV, blood vessel).

As our *in vitro* data suggested that human neutrophil bronchial-TEpM was dependent on ICAM-1 and independent of ICAM-2 expression, a mouse model of sterile inflammation was used to confirm these findings *in vivo*. Basal expression of ICAM-1 and ICAM-2 on naive murine lung epithelium were initially studied. ICAM-1 was expressed in blood vessels, as supported by earlier reports (27) and also strongly expressed on the alveolar epithelium and to a lesser extent on the bronchial epithelium (**Figure 5A**). ICAM-2 was found to be mildly expressed on the endothelium (**Figure 5B**), a previously known observation (27) and more strongly expressed on the bronchial epithelium than the alveolar epithelium (**Figure 5B**). Staining of naive murine lung with matched isotype control antibodies revealed minimal non-specific binding of ICAM-1 or ICAM-2 antibody (**Figures 5A, B**). Overall, ICAM-1 and ICAM-2 were differentially expressed on the alveolar or bronchial epithelium of naïve murine lungs, mirroring our earlier observations in normal human adult lung.

### Neutrophil Recruitment Into the Airways or Lungs Is Independent of ICAM-2 in an Animal Model of Sterile Inflammation

**Figure 6:**
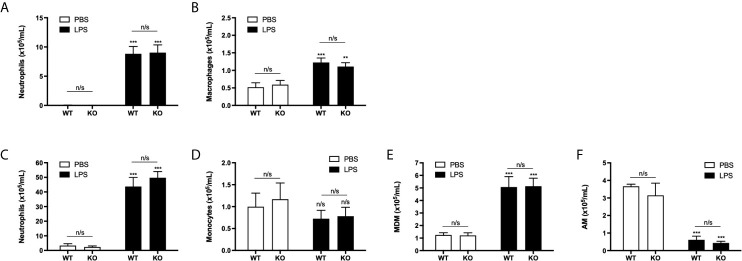
Neutrophil recruitment into the airways or lungs is independent of ICAM-2 during sterile inflammation *in vivo*. **(A, B)** ICAM-2 WT (*n*=8 per treatment group) or ICAM-2 KO (*n*=7 per treatment group) mice were challenged with 3.3 μg LPS or PBS *via* intranasal instillation for 24 h. Quantification of neutrophils or macrophages from recovered BALF was determined by cell counts from cytospins. **(C–F)** Quantification of recruited neutrophils, monocytes, monocyte-derived macrophage (MDM) or alveolar macrophages (AM) populations in the lung was determined from single cell lung homogenates by flow cytometric analysis. Any significant differences were determined using 2-way ANOVA statistical testing with Holm-Sidak post-hoc correction (n/s, not significant, **p < 0.01, ***p < 0.001). Asterisks above bars indicate significant differences compared to PBS-treated genotype unless stated otherwise.

To confirm our *in vitro* data showing neutrophil TEpM was ICAM-2 independent, the role of ICAM-2 in neutrophil TEpM was investigated *in vivo* using mice lacking global ICAM-2 expression in an established model of LPS-induced sterile inflammation model. Inhaled LPS can lead to neutrophil recruitment into the airways (28). Treatment of ICAM-2 WT or KO mice with inhaled LPS lead to significant recruitment of neutrophils and macrophages into the BALF compared to PBS-treated mice after 24 h (**Figures 6A, B**). However, there was no significant difference between LPS-treated ICAM-2 WT or KO mice (**Figures 6A, B**). In addition, neutrophils and monocyte-derived macrophages (MDMs) were significantly recruited into LPS-treated lungs compared to PBS treated controls (**Figures 6C, E**). Alveolar macrophages (AMs) were significantly reduced in LPS-treated lungs compared to PBS controls and numbers of monocytes were unchanged (**Figures 6D, F**). Similar to our observations in the BALF, there were no significant differences in recruited cells into the lung between LPS-treated ICAM-2 WT or KO mice (**Figures 6C–F**). Overall, this suggested that ICAM-2 is redundant in mediating neutrophil recruitment into the airways or lung *in vivo*.

### ICAM-1 Mediates Macrophage Recruitment Into the Lung but Is Not Required for Neutrophil TEpM Into the Airways *In Vivo*

**Figure 7:**
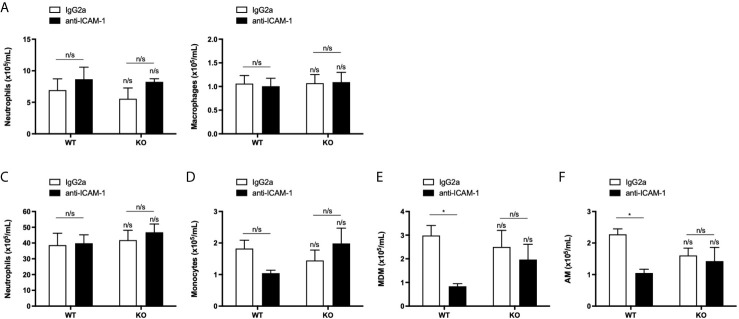
Neutrophil recruitment into the airways or lungs is independent of both LFA-1: ICAM-1 and ICAM-2 interactions during inflammation. **(A, B)** ICAM-2 WT or KO mice were pre-treated with 2 doses of 3 mg/kg IgG2a isotype control (*n*=4 WT or KO) or ICAM-1 blocking antibody (*n*=5 WT or KO) prior to intranasal challenge with 3.3 μg LPS for 24 h. Quantification of neutrophils or macrophages from recovered BALF was determined by cell counts from cytospins. **(C–F)** Quantification of recruited neutrophils, monocytes, monocyte-derived macrophages (MDM) or alveolar macrophages (AM) populations into the lung was determined from single cell lung homogenates by flow cytometric analysis. Any significant differences were determined using 2-way ANOVA statistical testing with Holm-Sidak post-hoc correction (n/s, not significant, *p < 0.05). Asterisks above bars indicate significant differences compared to PBS-treated genotype unless stated otherwise.

It has been reported, using KO mice, that LFA-1: ICAM-1 interactions mediate approximately 50% of neutrophil recruitment into the alveolar space during inflammation *in vivo* (29). To ensure that LFA-1:ICAM-1 interactions were not compensating for a lack of ICAM-2 in our animal model of sterile inflammation, ICAM-2 WT and KO mice were pre-treated with a blocking antibody directed against D1 of ICAM-1, the site for LFA-1 binding, before LPS challenge. Staining of murine lungs with an anti-isotype secondary antibody confirmed the ICAM-1 blocking antibody had reached the alveolar space (**Supplemental Figure 4**). Interestingly, LFA-1:ICAM-1 blockade did not significantly affect the number of neutrophils or macrophages recruited into the BALF of ICAM-2 WT or KO mice compared to isotype treated controls (**Figures 7A, B**). In addition, LFA-1:ICAM-1 blockade had no effect on neutrophil or monocyte recruitment to the lungs in either ICAM-2 WT or KO mice compared to isotype treated controls (**Figures 7C, D**). However, LFA-1:ICAM-1 blocking antibody treatment significantly reduced the numbers of recruited MDMs and AMs in the lungs of ICAM-2 WT mice (**Figures 7E, F**) but this effect was not seen in the ICAM-2 KO mice (**Figures 7E, F**). This suggested that although ICAM-1 and ICAM-2 do not mediate neutrophil recruitment, they may interact in a complex manner to mediate macrophage recruitment into inflamed lungs *in vivo*.

## Discussion

The pulmonary circulation is the largest vascular bed in the body and the lung contains a large marginated pool of neutrophils (30), ready to mount a rapid immune response against infection. Neutrophil recruitment during pulmonary inflammation is characterized by sequential steps from exiting the blood across the endothelium and into the lung interstitium (31). How neutrophils exit the lung across the epithelium is less well understood and the requirements for ICAM-1 or ICAM-2 in this process are ill-defined.

We found differential expression of ICAM-1 and ICAM-2 on healthy human and murine lung epithelium, with ICAM-1 expression more prominent on alveolar epithelium and more ICAM-2 found on the bronchial epithelium. ICAM-1 and ICAM-2 expression levels were also up-regulated in lungs from patients with inflammatory lung diseases associated with airway neutrophilia such as CF and to a lesser extent, COPD. These findings agreed with literature demonstrating up-regulation of ICAM-1 expression on bronchial epithelium during chronic inflammation (32, 33). Although, recent single-cell transcriptomics of healthy human lung have indicated that alveolar type I and II cells have the highest *icam1* expression (34). Human bronchial epithelial cells stimulated with TNF-α showed increased ICAM-2 mRNA and protein expression *in vitro*, although the evidence for ICAM-2 expression in the lung is conflicted. Our observation of strong bronchial epithelial ICAM-2 expression agrees with previous studies demonstrating ICAM-2 expression on human bronchial epithelial cells (21, 35). However, other studies have found no or very weak ICAM-2 expression in human lung epithelium (34, 36). This discrepancy with our results could be due to the lung tissue from heavy smokers used by González et al., which may affect ICAM-2 expression. It is still unclear why there is differential ICAM-1 and ICAM-2 expression on lung epithelium, one possible explanation could be regulated neutrophil exit from the interstitium for immune clearance by the mucociliary escalator *via* the bronchial epithelium (37) or to promote tissue repair *via* the alveolar epithelium (38, 39).

Whilst, the mechanisms of neutrophil trans-endothelial migration are well characterized, the role of cell adhesion molecules such as β2 integrins and ICAMs in neutrophil TEpM are ill-defined and sometimes contradictory. Tight epithelia are highly polarized and biochemical and imaging studies have shown that ICAM-1 is apically restricted on gut epithelial cells and does not play a role in leukocyte TEpM (40). The same was assumed to be true for the bronchial epithelium. However, we and others (41) have previously shown, with confocal and functional data, that although ICAM-1 and ICAM-2 are both polarized, both are expressed on the basolateral and to a greater extent, the apical surfaces, with a marked basal-to-apical gradient on bronchial epithelial cells (21). In this current work we show that neutrophil TEpM across a monolayer of differentiated human bronchial epithelial cells is ICAM-1 and β2-integrin dependent as supported by other studies (17–19) with no contribution from ICAM-2, despite high expression of this ligand.

Our previous work showed that lymphocyte migration across a bronchial epithelial monolayer is a multistep process dependent on LFA-1 adhesion to basolateral ICAM-1 and ICAM-2 and leukocyte migration along an ICAM gradient. This gradient guides lymphocytes into the paracellular space for negotiation of the tight junctions, so called ‘junctional recognition’. It is likely that ICAM-1 is playing a similar role in neutrophil TEpM across a ‘tight’ epithelium. This step may be less important in a leaky epithelium where junctional integrity has been compromised and crossing the junctions is no longer a rate-limiting step. This may explain the recent observation that when the interactions between LFA-1 on neutrophils and ICAM-1 on respiratory syncytial virus (RSV)-infected nasal airway epithelial cells were inhibited, this led to deceased neutrophil adhesion to the epithelial cells with no effect on subsequent neutrophil TEpM (42). We previously showed that apical ICAM-1 is not required for TEpM for lymphocytes but does play a role in leukocyte retention in the airway (21). Interestingly, here we also showed that neutrophil TEpM itself increases barrier permeability, perhaps in an ICAM-1/myosin light chain kinase dependent manner (43). This suggests that in the face of epithelial barrier disruption as may occur during overwhelming infection, inflammation or TEpM of large numbers of neutrophils, other cell adhesion molecules or extrinsic factors, such as chemokine gradients, may mediate the movement of neutrophils out into the airways.

The requirement for LFA-1: ICAM-1 in neutrophil recruitment into the airways and lung during LPS-induced pulmonary inflammation *in vivo* has been previously described (29, 44). These studies contrast with our findings where blockade of LFA-1: ICAM-1 in ICAM-2 wild-type mice did not significantly affect neutrophil recruitment into the lung or broncho-alveolar space after LPS challenge. These discrepancies in results could be due to our higher dose of LPS used to induce inflammation, which may disrupt the epithelial barrier (45), so reducing the requirement for ICAM-1 for TEpM. It should also be noted that our choice of ICAM-1 blocking antibody (clone KAT-1) has been shown to bind to the D-1 domain of ICAM-1 to prevent LFA-1 binding to ICAM-1 (46), therefore we cannot exclude the possibility that neutrophils may still be able to bind to domain 3 of ICAM-1 *via* Mac-1 in the presence of clone KAT-1 in our animal experiments. However, Basit et al., and Kumasaka et al., also used an ICAM-1 D-1 blocking antibody and have reached a different conclusion (29, 44). Work by Petrovich et al., has addressed this same question using ICAM-1^-/-^ mice to avoid the complications with ICAM-1 blocking antibodies. Despite their different approach and the absence of Mac-1:ICAM-1 interactions, they show, as we have here, that epithelial ICAM-1 is not required for TEpM in sterile inflammation (27).

The discrepancy between these *in vivo* results and the consistent *in vitro* demonstrations of a role for ICAM-1 in neutrophil TEpM across an intact bronchial epithelial barrier with high (> 2000 Ω) TEER support the concept that ICAM-1 may only regulate TEpM through an intact barrier or during milder inflammation. Furthermore it should also be noted that ICAM-1 may have other roles aside from cellular recruitment such as bacterial killing as seen in a pneumococcal infection model (although migration into the alveolar space was not assessed) (47) or activating NK cells to induce cell lysis during infection (48).

The role of ICAM-2 in mediating neutrophil TEpM has not been fully elucidated, although ICAM-2 facilitates lymphocyte (21) and eosinophil TEpM (15). We found very little requirement for ICAM-2 for neutrophil TEpM *in vitro* or *in vivo*, despite abundant expression on lung bronchial epithelium. In addition, we found the loss of ICAM-1 and ICAM-2 does not impact cellular recruitment into the airways or lung in response to pneumococcal bacterial infection (**Supplemental Figure 3**). Our results support Petrovich et al., who found that during strong inflammatory conditions such as LPS treatment when the epithelial barriers are disrupted, ICAM-1 and ICAM-2 are not required for neutrophil TEpM (27). In addition, Petrovich et al., also found significantly more neutrophils entrapped in the lungs of PBS-treated ICAM-1/ICAM-2 double knockout mice compared to PBS-treated wildtype controls and very few neutrophils recovered in the BAL (27). This would support our hypothesis that when the epithelial barrier is intact, ICAM-1 and/or ICAM-2 may be required for basal or constitutive trafficking of neutrophils out of the lung and into the airways.

As our *in vivo* data suggested that neutrophil recruitment into the lung or airways during inflammation is ICAM-1 and ICAM-2 independent, other adhesion molecules may play a more dominant role in neutrophil trafficking. One possibility is that neutrophil recruitment *in vivo* is driven by selectins such as E- and L-selectin (49). TREM-1 expressed on neutrophils has been shown to be required for TEpM during *P. aeruginosa* infection and for control of bacterial spread (50). However, the epithelial ligand for TREM-1 was not identified in this study. In addition, neutrophil TEpM has shown to be dependent on neutrophil expressed Mac-1 and JAM-A on the colonic epithelium (51), whether a similar mechanism also occurs in the lung is currently unknown.

It should also be noted that ICAM-1 and ICAM-2 may drive pulmonary recruitment of other leukocytes such as T cells. We have previously shown T cells use ICAM-1 and ICAM-2 for TEpM *in vitro* (21). However, ICAM-1 deficient mice exhibit reduced lymphocyte trapping in naive lung and additional ICAM-2 blockade did not further reduce lymphocyte numbers in the lung (52). It is still unknown the role these ligands play in lymphocyte TEpM during *in vivo* inflammation or infection. In addition, we showed ICAM-1 is required for MDM recruitment into inflamed lung as supported by *in vitro* studies demonstrating ICAM-1 dependent monocyte trans-endothelial migration (53). However, macrophages are still found in similar numbers in the lavage despite ICAM-1 blockade. ICAM-1 may play a role in leukocyte retention at the luminal epithelial surface, so that despite reduced numbers at this site; a higher percentage of cells are dislodged and found in the lavage. Intriguingly this effect is not seen in ICAM-2 KO mice, where ICAM-1 blockade has no effect on leukocyte recruitment to the interstitium. The fact that ICAM-2 deletion rescues the effect of ICAM-1 blockade may reflect the fact that monocytes also express ICAM-2 (7) required for cellular trafficking and this remains the focus of future work.

Overall, we have demonstrated for the first time, the differential expression of ICAM-1 and ICAM-2 on healthy adult human and murine lung epithelium and up-regulation of these ligands during inflammation and inflammatory lung diseases. Our studies demonstrated that neutrophil TEpM through an intact human bronchial epithelial cell monolayer is ICAM-1 and β2 integrin-dependent and neutrophil TEpM itself reduced bronchial epithelial barrier integrity. In addition, we have made the observation that neutrophil recruitment into the lung or airways is ICAM-2 independent during pulmonary sterile inflammation, and ICAM-2 deficiency is not compensated for by LFA-1: ICAM-1 interactions. This suggests that other mechanisms facilitate neutrophil exit into the airways in the absence of LFA-1: ICAM-1 and ICAM-2 during an inflammatory response.

## Data Availability Statement

The original contributions presented in the study are included in the article/**Supplementary Material**. Further inquiries can be directed to the corresponding author.

## Ethics Statement

The studies involving human participants were reviewed and approved by UK National Research Ethics Committee (13/LO/0900). The patients/participants provided their written informed consent to participate in this study. The animal study was reviewed and approved by University College London and the UK Home Office.

## Author Contributions

JP and CS conceived and supervised the study. CR, DC, CS, and JP designed experiments. CR and DC performed and analysed the experiments. RJ, AW, JB, and CS helped perform some of the animal experiments. All authors contributed to the article and approved the submitted version.

## Funding

This work was undertaken at UCLH/UCL, which received a proportion of the funding from the UK’s Department of Health’s NIHR Biomedical Research Centre’s fund scheme. This work was also supported by the Medical Research Council (MRC) (grant codes G0800340 and MR/K004158/1) and a European Commission FP7 award PIEF GA-2012-326928 to CR.

## Conflict of Interest

The authors declare that the research was conducted in the absence of any commercial or financial relationships that could be construed as a potential conflict of interest.

## Publisher’s Note

All claims expressed in this article are solely those of the authors and do not necessarily represent those of their affiliated organizations, or those of the publisher, the editors and the reviewers. Any product that may be evaluated in this article, or claim that may be made by its manufacturer, is not guaranteed or endorsed by the publisher.
