# Supplementary material for: ICAM-1 and ICAM-2 Are Differentially Expressed and Up-Regulated on Inflamed Pulmonary Epithelium, but Neither ICAM-2 nor LFA-1: ICAM-1 Are Required for Neutrophil Migration Into the Airways In Vivo
Source: Front Immunol. 2021 Aug 16;12:691957. doi: 10.3389/fimmu.2021.691957 (PMC8415445; doi:10.3389/fimmu.2021.691957)
Supplement: Supplementary file 1 [file Image_1.pdf]

Supplemental Figure 1. FACS gating strategy for counting trans-epithelial migrated neutrophil *in vitro*.

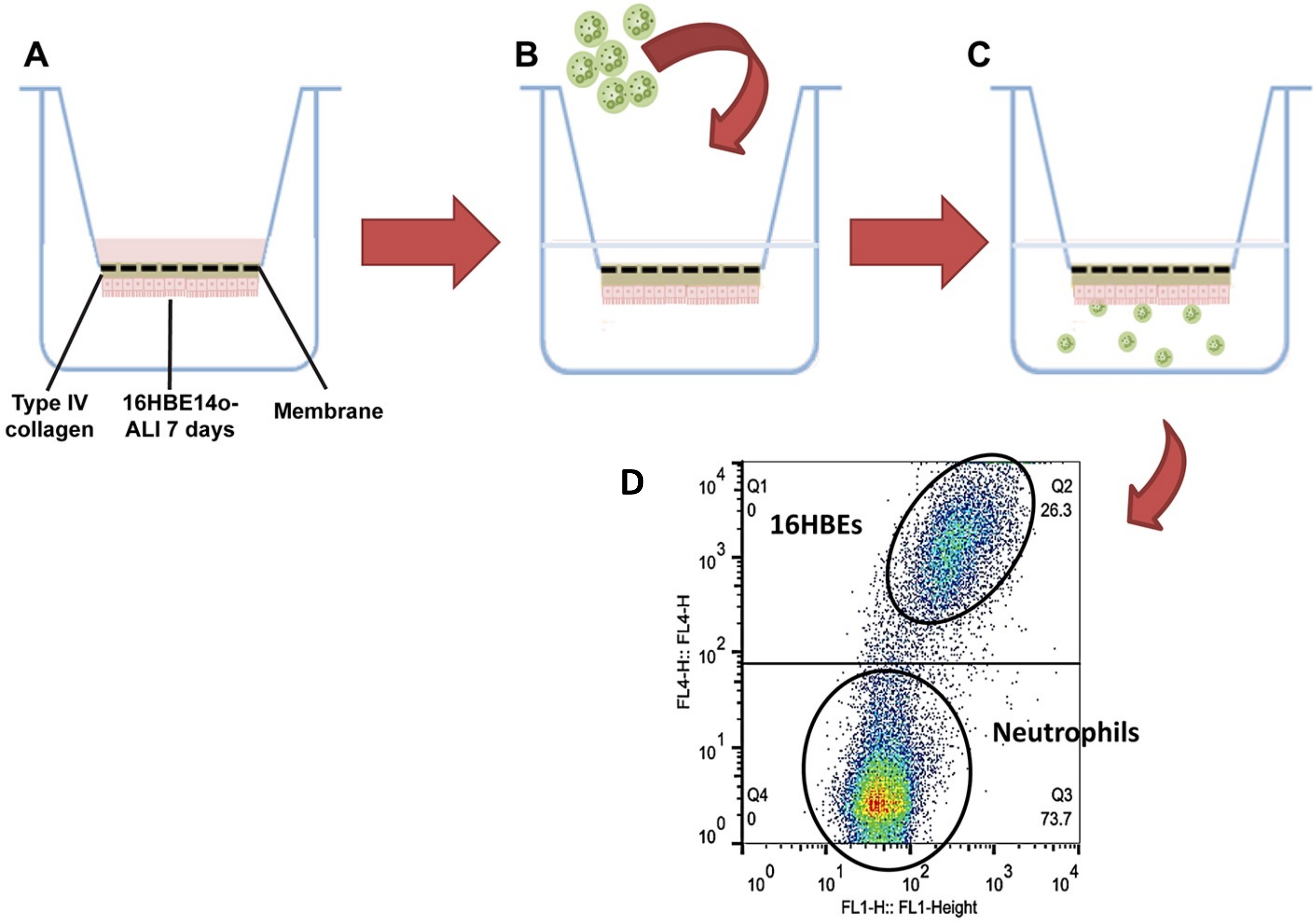

A. Schematic of DiD-labelled 16HBE14o- monolayers grown at Air Liquid Interface (ALI) using a trans-well *in vitro* system.

B. CMFDA-labelled neutrophils isolated from human peripheral blood were added to the top of trans-well insert and the chemoattractant, fMLP added to the bottom of the well

C. Neutrophils were allow to migrate towards fMLP in the bottom of the well for 3 h.

D. Cells were detached from the bottom of the trans-well upon the addition of 0.5 M EDTA and run through a FACS machine to distinguish between DiD-labelled 16HBE cells ($FL4^{+}$) and CMFDA-labelled neutrophils ($FL1^{+}$). The number of CMFDA-labelled neutrophils was counted and used to calculate percentage of TEpM relative to the fMLP positive control.
