# Supplementary material for: ICAM-1 and ICAM-2 Are Differentially Expressed and Up-Regulated on Inflamed Pulmonary Epithelium, but Neither ICAM-2 nor LFA-1: ICAM-1 Are Required for Neutrophil Migration Into the Airways In Vivo
Source: Front Immunol. 2021 Aug 16;12:691957. doi: 10.3389/fimmu.2021.691957 (PMC8415445; doi:10.3389/fimmu.2021.691957)
Supplement: Supplementary file 2 [file Image_2.pdf]

Supplemental Figure 2. FACS gating strategy for distinguishing recovered immune cells post *in vivo* challenge.

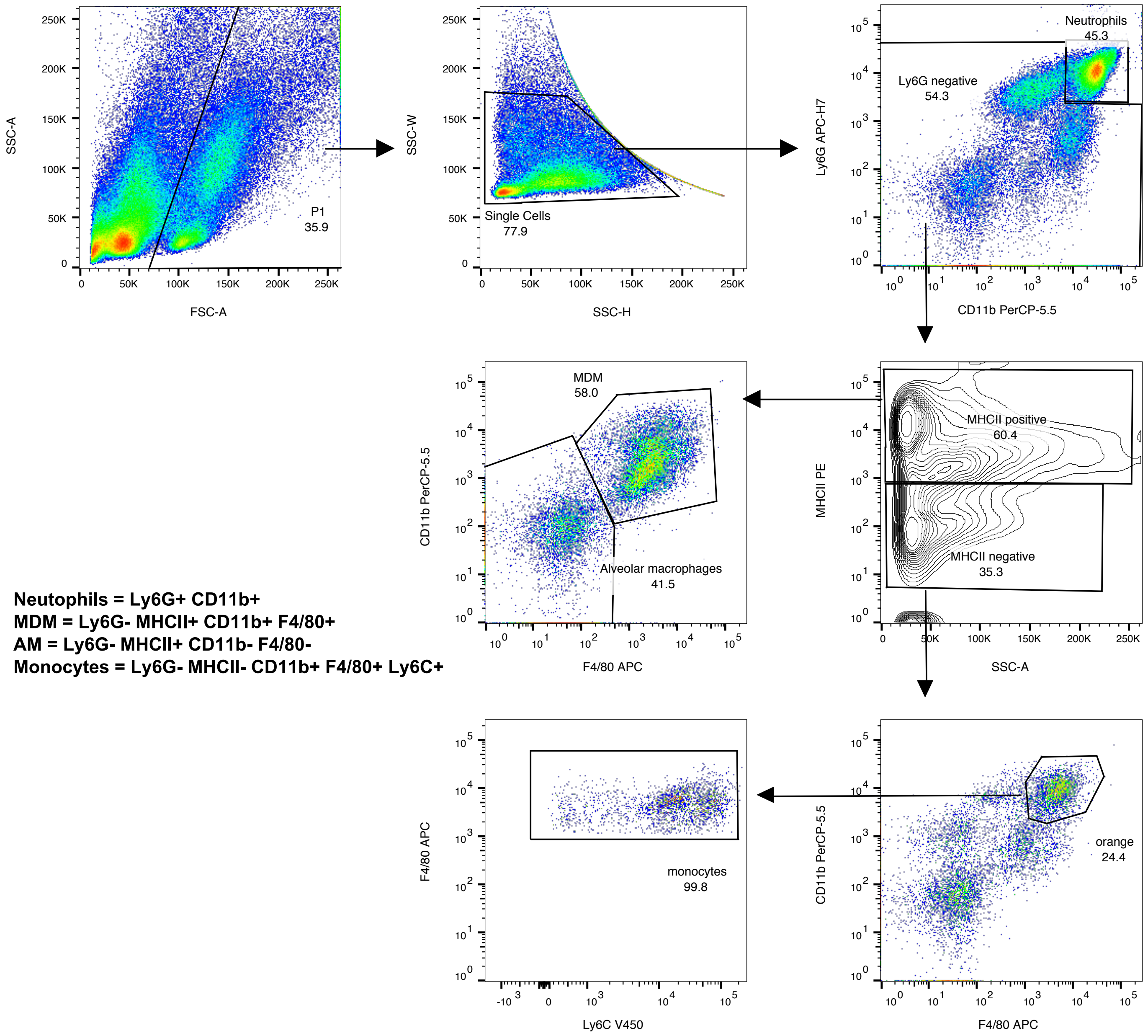

Single cell lung homogenates from challenged mice were stained with a panel of antibodies (Ly6G-APC-Cy7, Ly6C-V450, CD11b-PerCP-5.5, CD11c-PE-Cy7, MHCII-PE or F4/80-APC) to determine various recruited cellular populations. We used the above gating strategy to distinguish between neutrophil, monocyte-derived macrophages (MDM), alveolar macrophages (AM) and monocytes populations.
