# Supplementary material for: ICAM-1 and ICAM-2 Are Differentially Expressed and Up-Regulated on Inflamed Pulmonary Epithelium, but Neither ICAM-2 nor LFA-1: ICAM-1 Are Required for Neutrophil Migration Into the Airways In Vivo
Source: Front Immunol. 2021 Aug 16;12:691957. doi: 10.3389/fimmu.2021.691957 (PMC8415445; doi:10.3389/fimmu.2021.691957)
Supplement: Supplementary file 3 [file Image_3.pdf]

Supplemental Figure 3. Neutrophil recruitment into the airways or lungs is independent of ICAM-1:LFA-1 or ICAM-2 interactions during pneumococcal infection.

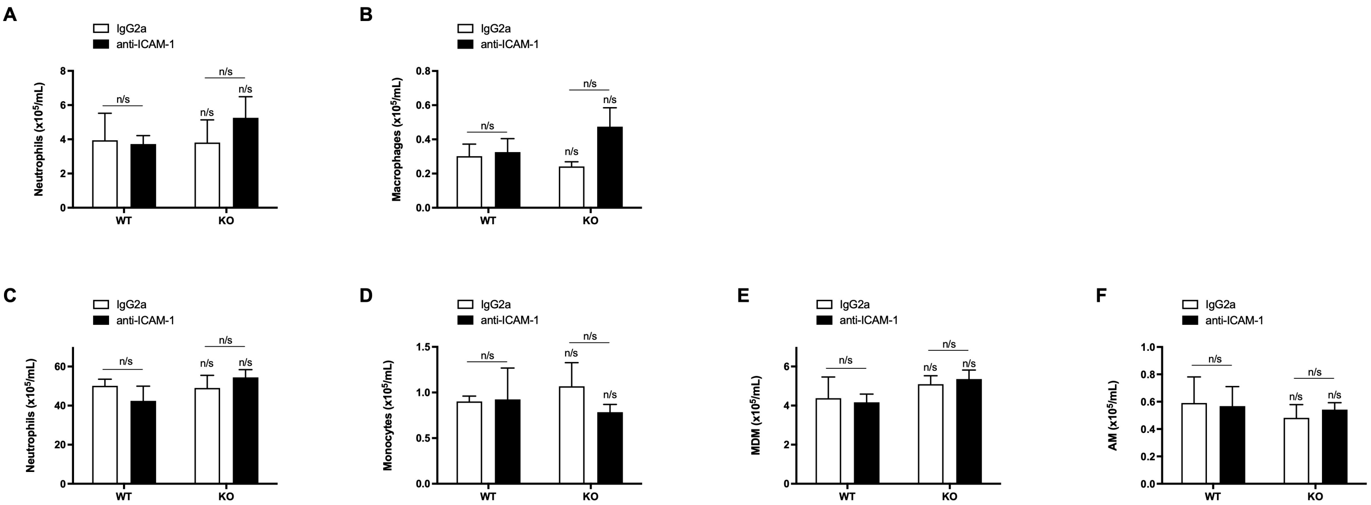

WT or KO mice (*n*=5-6 per group) were pre-treated with 2 intraperitoneal doses of 75 μg IgG2a isotype control or ICAM-1 blocking antibody (clone KAT-1) prior to intranasal infection with *S. pneumoniae* (19F isolate, 5x10^6 CFU/mouse) for 24 h.

A-B. Quantification of neutrophils or macrophages from recovered BALF was determined by cell counts from cytospins.
C-F. Quantification of recruited neutrophils, monocytes, monocyte-derived macrophages (MDM) or alveolar macrophages (AM) populations into the lung was determined from single cell lung homogenates by flow cytometric analysis.

No significant differences between treatment groups were found using 2-way ANOVA with Holm-Sidak post-hoc correction (n/s = not significant). Asterisks above bars indicate significant differences compared to PBS treated genotype unless stated otherwise.
