# Supplementary material for: ICAM-1 and ICAM-2 Are Differentially Expressed and Up-Regulated on Inflamed Pulmonary Epithelium, but Neither ICAM-2 nor LFA-1: ICAM-1 Are Required for Neutrophil Migration Into the Airways In Vivo
Source: Front Immunol. 2021 Aug 16;12:691957. doi: 10.3389/fimmu.2021.691957 (PMC8415445; doi:10.3389/fimmu.2021.691957)
Supplement: Supplementary file 4 [file Image_4.pdf]

Supplemental Figure 4. Penetration of blocking ICAM-1 antibody into murine lungs.

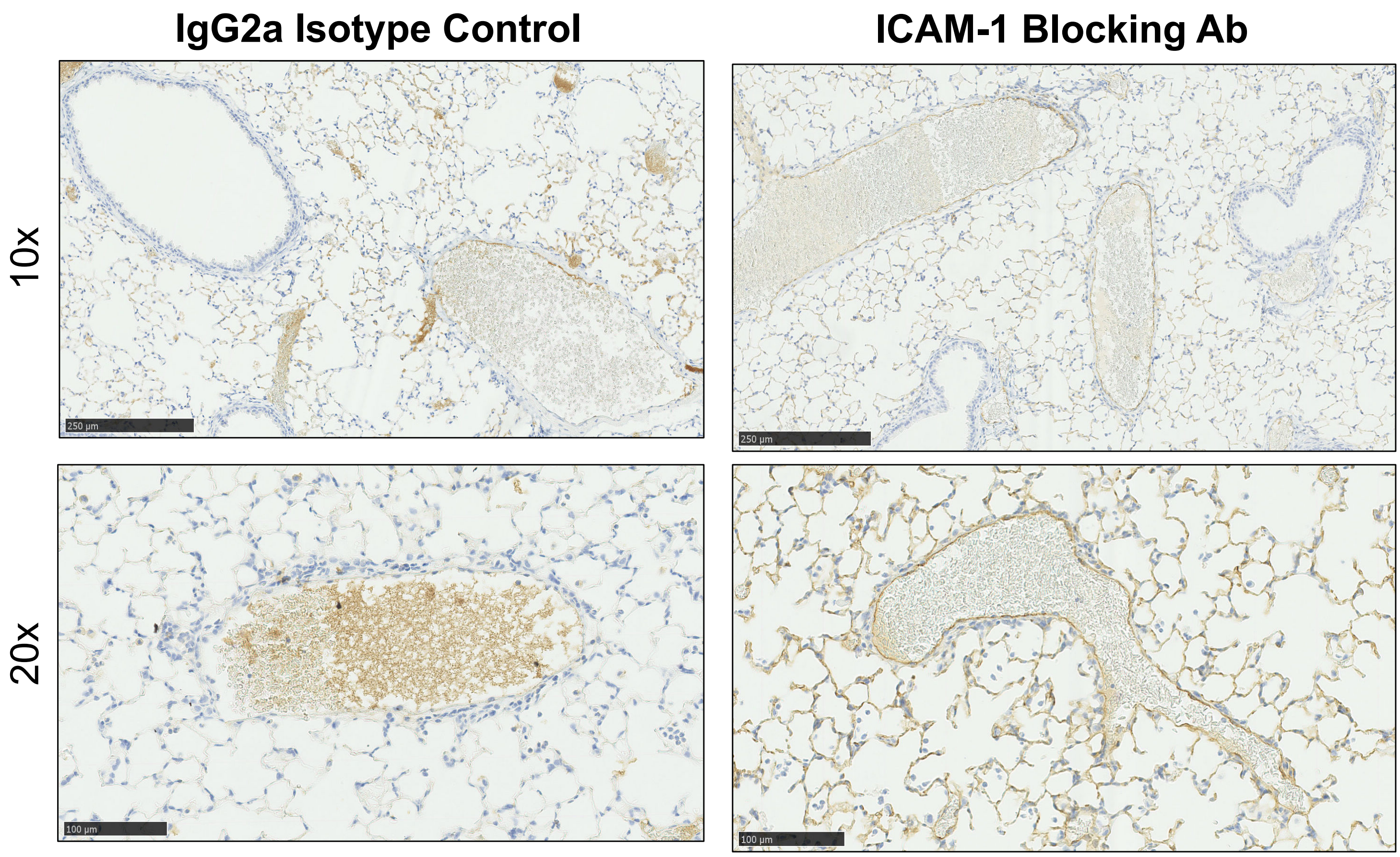

Mice were pre-treated with 2 doses of 75 µg IgG2a isotype control or ICAM-1 blocking antibody (clone KAT-1) via an intra-peritoneal route prior to intranasal challenge with 3.437 µg LPS for 24 h.

Confirmation that ICAM-1 blocking antibody or matched isotype control had reached the lung was achieved by staining lung sections from treated mice with rabbit anti-rat secondary antibody and developing with DAB (brown staining), followed by nuclear counter-staining. Images are shown at 10x and 20x objective as denoted by the scale bar.
